# A dynamic attribute reduction algorithm based on relative neighborhood discernibility degree

**DOI:** 10.1038/s41598-024-66264-x

**Published:** 2024-07-08

**Authors:** Weibing Feng, Tiantian Sun

**Affiliations:** College of Science, Xi’an University of Science and Technology, Xi’an, China

**Keywords:** Attribute reduction, Incremental update mechanism, Relative neighborhood discernibility degree, Weakly labeled data, Engineering, Mathematics and computing

## Abstract

This paper addresses the current existence of attribute reduction algorithms for incomplete hybrid decision-making systems, including low attribute reduction efficiency, low classification accuracy and lack of consideration of unlabeled data types. To address these issues, this paper first redefines the weakly labeled relative neighborhood discernibility degree and develops a non-dynamic attribute reduction algorithm. In addition, this paper proposes an incremental update mechanism for weakly tagged relative neighborhood discernibility degree and introduces a new dynamic attribute reduction algorithm for increasing the set of objects based on it. Meanwhile, this paper also compares and analyses the improved algorithm proposed in this study with two existing attribute reduction algorithms using 8 data sets in the UCI database. The results show that the dynamic attribute reduction algorithm proposed in this paper achieves higher attribute reduction efficiency and classification accuracy, which further validates the effectiveness of the algorithm proposed in this paper.

## Introduction

Attribute reduction^1–8^, also known as feature selection, has garnered significant attention in the current era of continuous data expansion. It plays a crucial role in machine learning and data mining by aiming to diminish complexity and redundancy in data. This is achieved through the elimination of unnecessary or redundant attributes, simplifying the dataset and improving analysis efficiency. Consequently, numerous methods for attribute reduction have been developed and extensively utilized^9–19^.

In recent years, scholars have conducted in-depth research on the attribute reduction of traditional rough sets^20–22^ and have proposed many efficient algorithms for attribute reduction. However, traditional attribute reduction algorithms are only suitable for static datasets. In today’s big data environment, data in practical applications is often dynamic, resulting in a common phenomenon of increasing the object set. As a result, scholars have conducted research on attribute reduction for changes in the object set. Shu et al.^23^ proposed a dependency-based dynamic attribute reduction algorithm for incomplete systems, while Wei et al.^24^ in their research on symbolic data, proposed a discriminative matrix-based algorithm and constructed a dynamic attribute reduction algorithm. Moreover, Yang et al.^25^ proposed a dynamic attribute reduction algorithm based on relative discriminative relationships in fuzzy rough sets, and Liang et al.^26^ proposed a dynamic attribute reduction algorithm based on information entropy for discrete data. Xiang^27^ further contributed to the field by proposing a dynamic attribute reduction algorithm that increases object sets for neighborhood systems, and Sheng et al.^28^ came up with a dynamic attribute reduction algorithm based on neighborhood discernibility degree for mixed data. These research efforts have significantly advanced the methodology and algorithms available for dynamic attribute reduction, addressing the challenges posed by the increasing object set in practical big data applications.

However, the aforementioned dynamic attribute reduction algorithm can only handle labeled data sets and does not consider unlabeled data types. In real-world applications, data often contains missing or omitted information due to factors such as data collection methods, human resources, and material resources. This leads to incomplete and unlabeled data, making dynamic attribute reduction in weakly labeled incomplete decision systems an important issue. However, there is limited research on this aspect by scholars.

Relative neighborhood discernibility degree can not only perform attribute reduction for labeled data, but also in unlabeled datasets, relative neighborhood discernibility degree can also perform attribute reduction for data without restriction to labeled datasets. Moreover, relative neighborhood discernibility degree has the features of high accuracy, adaptability, efficiency and robustness, and has a wide application prospect in the field of attribute reduction. Its advantages make it better able to meet the needs of users and improve the quality and effect of data when dealing with data in practical applications. Therefore, this paper proposes an improved dynamic attribute reduction algorithm based on weak label relative neighborhood discernibility degree to address this problem.

In reference^29^, an attribute reduction algorithm for incomplete mixed decision systems was given without providing an attribute reduction algorithm for dynamic changes in the object set and weakly labeled data type. Thus, inspired by reference^29^, this study improves the definition of weakly labeled relative neighborhood discernibility degree and proposes a non-dynamic attribute reduction algorithm for weakly labeled incomplete mixed decision systems. Furthermore, an incremental update mechanism for weakly labeled relative neighborhood discernibility degree is presented to address the addition of object sets and a dynamic attribute reduction algorithm is designed. Finally, experimental analysis on datasets from the UCI database verifies the higher efficiency of the proposed dynamic attribute reduction algorithm.

This article introduces several innovations: (1) a refinement of the definition of weakly labeled relative neighborhood discernibility degree based on relative neighborhood discernibility degree; (2) a non-dynamic attribute reduction algorithm for weakly labeled incomplete mixed decision systems; (3) the development of an incremental update mechanism for increasing object sets; (4) an dynamic attribute reduction algorithm for weakly labeled incomplete mixed decision systems with an increased object set.

The article is structured as follows: Section 2 introduces the basic concepts of rough sets and weakly-labeled incomplete mixed decision systems. Following this, Section 3 presents the non-dynamic attribute reduction algorithm for weakly-labeled incomplete mixed decision systems. In Section 4, the incremental update mechanism for increasing the object set with relative neighborhood discernibility degree and dynamic attribute reduction algorithm for weakly labeled incomplete mixed decision systems is described. Section 5 is dedicated to the presentation of the experimental analysis, and Section 6 summarizes and introduces future research directions.

## Basic knowledge

In this section, we will cover the fundamental concepts of weakly labeled incomplete mixed decision systems and rough sets. For further details, please refer to reference^35–37^.

Definition 1^30^

$WDIS = \left\langle {U,C \cup D,V,f} \right\rangle$ is used to represent a weakly labeled incomplete mixed decision system; where $U = \left\{ {x_{1} ,x_{2} , \cdots ,x_{n} } \right\}$ represents a non-empty finite set of objects, also called the universe;$C$ represents a non-empty finite set of attributes,$C = C_{d} \cup C_{r}$,$C_{d}$ represents a discrete set of attributes,$C_{r}$ represents a continuous set of attributes,$D$ is decision attribute,$C \cup D = \emptyset$,$V = \mathop \cup \limits_{a \in C} V_{a}$,$V_{a}$ is a set of all possible values of attributes $a \in C$;$f$ denotes the mapping function of $U \times C \to V$,which assigns a value to each object’s attributes, means $\forall a \in C$,$x \in U$,$f(x,a) \in V_{a}$,and at least one attribute $a \in C$,with $f(a,x) = *$,that is the attribute has missing values. Among them, there are also missing values for the decision attribute $d$,that is,$\exists x_{i} \in U$,with $d(x_{i} ) = *$,which represents the set of unlabeled objects. Therefore $U = M \cup L$,where $M$ represents the set of unlabeled objects and $L$ represents the set of labeled objects.

Table 1 shows a weakly labeled incomplete mixed decision system, $U = \left\{ {x_{1} ,x_{2} , \cdots ,x_{7} } \right\}$ is a domain, $L = \left\{ {x_{1} ,x_{2} ,x_{3} ,x_{4} } \right\}$ is a set of labeled objects, and $M = \left\{ {x_{5} ,x_{6} ,x_{7} } \right\}$ is a set of unlabeled objects; $C = \left\{ {c_{1} ,c_{2} , \cdots ,c_{5} } \right\}$ is an attribute set, $C_{d} = \left\{ {c_{1} ,c_{2} ,c_{3} } \right\}$ is a discrete attribute set, $C_{r} = \left\{ {c_{4} ,c_{5} } \right\}$ is a continuous attribute set,$D$ is decision attributes.

**Table 1:** Weakly labeled incomplete mixed decision systems.

$U$	$c_{1}$	$c_{2}$	$c_{3}$	$c_{4}$	$c_{5}$	$D$
$x_{1}$	1	2	2	4.67	2.56	1
$x_{2}$	$*$	2	$*$	4.32	2.43	1
$x_{3}$	2	$*$	1	4.41	$*$	0
$x_{4}$	1	2	$*$	4.08	3.50	0
$x_{5}$	1	2	2	$*$	3.57	$*$
$x_{6}$	2	$*$	2	4.52	$*$	$*$
$x_{7}$	1	1	2	$*$	1.12	$*$

### Definition 2^31^

Given a weakly labeled incomplete mixed decision system $WDIS = \left\langle {U,C \cup D,V,f} \right\rangle$,where $B \subseteq C$ and $B = B_{d} \cup B_{r}$,$B_{d}$ represents a discrete attribute set,$B_{r}$ represents a continuous attribute set,$\forall a \in B$,the neighborhood tolerance relationship of the attribute set $B$ and the neighborhood tolerance class of $x_{i}$ with respect to attribute set $B$ are defined as:

1 $$NT_{B}^{\delta } = \left\{ \begin{gathered} (x_{i} ,x_{j} ) \in U^{2} :(\forall a \in B_{d} ,f(a,x_{i} ) = * \vee f(a,x_{j} ) = * \vee f(a,x_{i} ) = f(a,x_{j} )) \hfill \\ \wedge (\forall a \in B_{r} ,f(a,x_{i} ) = * \vee f(a,x_{j} ) = * \vee |f(a,x_{i} ) - f(a,x_{j} )| \le \delta ) \hfill \\ \end{gathered} \right\}$$

2 $$NT_{B}^{\delta } (x_{i} ) = \left\{ {x_{j} \in U|(x_{i} ,x_{j} ) \in NT_{B}^{\delta } } \right\}$$

where $\delta$ is the neighborhood radius, which is a non-negative constant.

For example, in Table 1$NT_{{c_{1} }}^{\delta } (x_{1} ) = \{ x_{1} ,x_{2} ,x_{4} \}$;$NT_{{c_{1} }}^{\delta } (x_{2} ) = \{ x_{1} ,x_{2} ,x_{3} ,x_{4} \}$;$NT_{{c_{{}} }}^{\delta } (x_{3} ) = \{ x_{2} ,x_{3} \}$;

$$NT_{{c_{1} }}^{\delta } (x_{4} ) = \{ x_{1} ,x_{2} ,x_{4} \}$$;$$NT_{{c_{1} }}^{\delta } (x_{5} ) = \{ x_{5} ,x_{7} \}$$;$$NT_{{c_{1} }}^{\delta } (x_{6} ) = \{ x_{6} \}$$;$$NT_{{c_{1} }}^{\delta } (x_{7} ) = \{ x_{5} ,x_{7} \}$$.

### Definition 3

^28^:Given a weakly labeled incomplete mixed decision system $WDIS = \left\langle {U,C \cup D,V,f} \right\rangle$.In the unlabeled set of objects $x_{i} \subseteq M$,where $B \subseteq C$,then the relative neighbor-hood discernibility degree of $B$ under $M$ is:

3 $$NDD_{B}^{M} (C) = \sum\limits_{i = 1}^{|M|} {|NT_{B}^{\delta } (x_{i} )|} - \sum\limits_{i = 1}^{|M|} {|NT_{C}^{\delta } (x_{i} )|}$$

Note:$$NDD_{B}^{M} (C) = 0$$ when $$B = C$$.

### Definition 4

^29^:Given a weakly labeled incomplete mixed decision system $WDIS = \left\langle {U,C \cup D,V,f} \right\rangle$.In the set of labeled objects $x_{i} \subseteq L$,where $B \subseteq C$,the decision class of $x_{i}$ in $D$ is $\left[ {x_{i} } \right]_{D}$,then the relative neighborhood discernibility degree of $B$ under $L$ is:

4 $$NDD_{B}^{L} (D) = \sum\limits_{i = 1}^{|L|} {|NT_{B}^{\delta } (x_{i} )|} - \sum\limits_{i = 1}^{|L|} {|NT_{B}^{\delta } (x_{i} ) \cap \left[ {x_{i} } \right]_{D} |}$$

## Non-dynamic attribute reduction algorithm for a weakly labeled incomplete mixed decision system

### Non-dynamic attribute reduction algorithm

The definitions 3 and 4 above provide definitions for unlabeled and labeled relative neighborhood discernibility degree. Building on definitions 3 and 4, this section improves the definition of weakly labeled relative neighborhood discernibility degree, laying the foundation for proposing a new algorithm for non-dynamic attribute reduction in weakly labeled incomplete decision systems.

#### Definition 5

Given a weakly labeled incomplete mixed decision system $WDIS = \left\langle {U,C \cup D,V,f} \right\rangle$,where $B \subseteq C$,then the relative neighborhood discernibility degree of $B$ under $U$ is:

5 $$NDD_{B} (U) = NDD_{B}^{M} (C) + NDD_{B}^{L} (D)$$

Special attention:$NDD_{B}^{M} (C) = 0$ when $M = \emptyset$;$NDD_{B}^{L} (D) = 0$ when $L = \emptyset$.

#### Theorem 1

Given a weakly labeled incomplete mixed decision system $WDIS = \left\langle {U,C \cup D,V,f} \right\rangle$,where $B_{1} \subseteq B_{2} \subseteq C$, then: $NDD_{{B_{1} }} (U) \ge NDD_{{B_{2} }} (U)$.

#### Proof

From Definition 5, we can directly obtain:

$$\begin{gathered} NDD_{{B_{1} }} (U) - NDD_{{B_{2} }} (U) \hfill \\ { = }NDD_{{B_{{1}} }}^{M} (C) + NDD_{{B_{{1}} }}^{L} (D){ + }NDD_{{B_{{2}} }}^{M} (C) + NDD_{{B_{{2}} }}^{L} (D) \hfill \\ = (\sum\limits_{i = 1}^{|M|} {|NT_{{B_{1} }}^{\delta } (x_{i} )|} - \sum\limits_{i = 1}^{|M|} {|NT_{C}^{\delta } (x_{i} )|} ) - (\sum\limits_{i = 1}^{|M|} {|NT_{{B_{2} }}^{\delta } (x_{i} )|} - \sum\limits_{i = 1}^{|M|} {|NT_{C}^{\delta } (x_{i} )|} ) + (\sum\limits_{i = 1}^{|L|} {|NT_{{B_{1} }}^{\delta } (x_{i} )|} - \sum\limits_{i = 1}^{|L|} {|NT_{{B_{2} }}^{\delta } (x_{i} )|} ) \hfill \\ - (\sum\limits_{i = 1}^{|L|} {|NT_{{B_{1} }}^{\delta } (x_{i} ) \cap [x_{i} ]_{D} |} - \sum\limits_{i = 1}^{|L|} {|NT_{{B_{2} }}^{\delta } (x_{i} ) \cap [x_{i} ]_{D} |} ) \hfill \\ \end{gathered}$$

Due to $B_{1} \subseteq B_{2}$,then $\forall x_{i} \in U$ resulting in satisfaction $NT_{{B_{2} }}^{\delta } (x_{i} ) \subseteq NT_{{B_{1} }}^{\delta } (x_{i} )$.

The conclusion reached is that $NDD_{{B_{1} }} (U) \ge NDD_{{B_{2} }} (U)$.

The theorem 1 mentioned above shows that as the attribute set increases, the relative neighborhood discernibility degree of weak labeling does not increase monotonically. This provides a theoretical basis for attribute reduction in incomplete decision systems with weak labeling. Therefore, based on the monotonicity of relative neighborhood discernibility degree of weak labeling, a non-dynamic attribute reduction algorithm can be constructed.

#### Definition 6

Given a weakly labeled incomplete mixed decision system $WDIS = \left\langle {U,C \cup D,V,f} \right\rangle$,$\forall a \in B \subseteq C$,the significance of the internal attributes of $a$ is:

6 $$sig^{inner} (a,B) = NDD_{{B - \left\{ a \right\}}} (U) - NDD_{B} (U)$$

#### Definition 7

Given a weakly labeled incomplete mixed decision system $WDIS = \left\langle {U,C \cup D,V,f} \right\rangle$,$\forall a \in C - B$,the significance of the external attributes of $a$ is:

7 $$sig^{outer} (a,B) = NDD_{B} (U) - NDD_{{B \cup \left\{ a \right\}}} (U)$$

Note: When $B = \emptyset$,$NDD_{B}^{M} (C) = |M|^{2}$,$NDD_{B}^{L} (D) = |L|^{2}$,then $NDD_{B} (U) = |M|^{2} + |L|^{2}$.

#### Definition 8

Given a weakly labeled incomplete mixed decision system $WDIS = \left\langle {U,C \cup D,V,f} \right\rangle$,if $R \subseteq C$ is an attribute reduction set, then $R$ satisfies:

8 $$\begin{gathered} (1)NDD_{R} (U) = NDD_{C} (U); \hfill \\ (2)\forall a \in R,NDD_{R} (U) \le NDD_{{R - \left\{ a \right\}}} (U). \hfill \\ \end{gathered}$$

The literature^22^ presents a dynamic attribute reduction algorithm based on discriminant matrix for increasing the object set; whereas, the literature^24^ presents a dynamic attribute reduction algorithm for increasing the object set based on information entropy. However, these algorithms are only designed for single, labeled data type attribute reduction, and are not applicable for mixed or unlabeled data. To address this issue, this paper proposes a non-dynamic attribute reduction algorithm for weakly labeled incomplete mixed decision systems based on weak label relative neighborhood discernibility degree, as shown in Algorithm 1.

**Algorithm 1:**
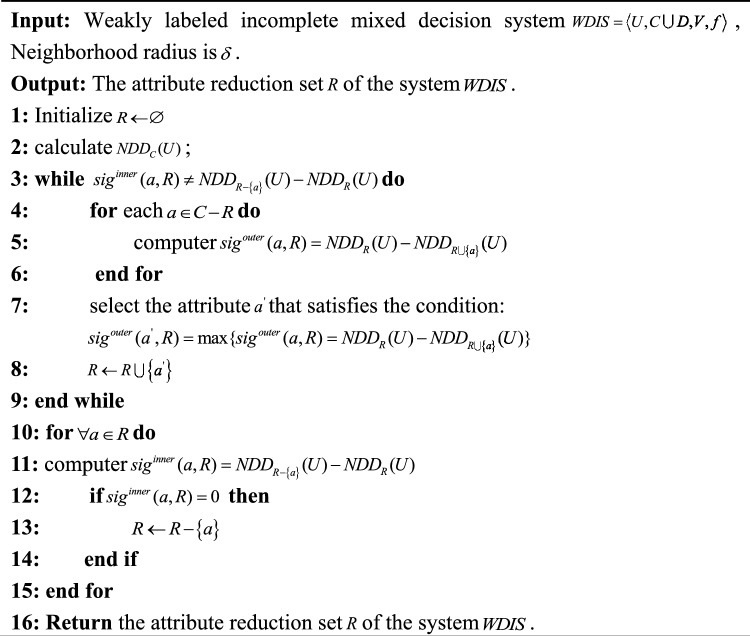
A Non dynamic attribute reduction algorithm for weakly labeled incomplete mixed decision systems.

Time complexity of non-dynamic Algorithm 1: Step 1 calculates the relative neighborhood discernibility degree of the entire set of attributes, whose time complexity is $O(|U||C|)$. Step 2 selects the most important attributes in each loop to be added to the set of candidate attributes until the termination condition is satisfied, whose time complexity is $O(|U|^{2} |C|^{3} )$, and Step 5 removes redundant attributes from the set of candidate attributes, whose time complexity is $O(|U^{2} ||C|^{2} )$. Therefore, the time complexity of Algorithm 1 is $O(|U|^{2} |C|)^{3}$.

### Example analysis

To further elaborate on the weakly labeled incomplete mixed decision system’s non-dynamic attribute reduction algorithm 1 proposed in this paper, the feasibility of the non-dynamic attribute reduction algorithm 1 is verified using the data in Table 2 as an example, with a neighborhood radius of $\delta = 0.2$.

**Table 2:** Weakly labeled incomplete mixed decision systems.

$U$	$C_{1}$	$C_{2}$	$C_{3}$	$C_{4}$	$C_{5}$	$D$
$x_{1}$	$b$	$a$	$a$	1.12	1.11	1
$x_{2}$	$*$	$a$	$*$	1.35	1.13	1
$x_{3}$	$a$	$*$	$b$	2.41	1.12	0
$x_{4}$	$b$	$a$	$a$	1.41	2.50	0
$x_{5}$	$b$	$a$	$a$	$*$	2.57	1
$x_{6}$	$a$	$b$	$a$	1.12	2.23	$*$
$x_{7}$	$b$	$b$	$a$	$*$	1.12	$*$

Table 2 represents a weakly labeled incomplete mixed decision system, where $U = \left\{ {x_{1} ,x_{2} ,...,x_{7} } \right\}$,$L = \left\{ {x_{1} ,x_{2} ,x_{3} ,x_{4} ,} \right.\left. {x_{5} } \right\}$ is a set of labeled objects and $M = \left\{ {x_{6} ,x_{7} } \right\}$ is a set of unlabeled objects;$C = \left\{ {C_{1} ,C_{2} , \cdots C_{5} } \right\}$ is attribute set,$C_{d} = \left\{ {C_{1} ,C_{2} ,C_{3} } \right\}$ is discrete attribute set,$C_{r} = \left\{ {C_{4} ,C_{5} } \right\}$ is continuous attribute set;$D$ is decision attributes. From Table 2 it follows that the neighborhood tolerance classes of $x_{i}$ with respect to $C$ is: $NT_{C}^{\delta } (x_{1} ) = \{ x_{1} \}$;$NT_{C}^{\delta } (x_{2} ) = \{ x_{2} \}$;$NT_{C}^{\delta } (x_{3} ) = \{ x_{3} \}$;$NT_{C}^{\delta } (x_{4} ) = \{ x_{4} ,x_{5} \}$;$NT_{C}^{\delta } (x_{5} ) = \{ x_{4} ,x_{5} \}$;$NT_{C}^{\delta } (x_{6} ) = \{ x_{6} \}$;$NT_{C}^{\delta } (x_{7} ) = \{ x_{7} \}$.In labeled datasets, the decision-making categories for $x_{i}$ under $D$ is:$[x_{1} ]_{D} = [x_{2} ]_{D} = [x_{5} ]_{D} = \{ x_{1} ,x_{2} ,x_{5} \}$;$[x_{3} ]_{D} = [x_{4} ]_{D} = \{ x_{3} ,x_{4} \}$.According to step 1: Command $R = \emptyset$,calculated $NDD_{C} (U) = 2$.According to step 2:$\forall c \in C - R$,the calculated significance of external attributes is:$sig^{outer} (c_{1} ,R) = 21$,$sig^{outer} (c_{2} ,R) = 15$ ,$sig^{outer} (c_{3} ,R) = 19$,$sig^{outer} (c_{4} ,R) = 21$,$sig^{outer} (c_{5} ,R) = 23$.According to step 3: Identify the attribute in $C - R$ with the highest significance for external attributes is $c_{5}$,then $R = R \cup \left\{ {c_{5} } \right\}$,where $NDD_{R} (U) = 6$.

According to step 4:$NDD_{R} (U) \ne NDD_{C} (U)$,go to step 2; Continuing to calculate:$\forall c \in C - R$,the calculated significance of external attributes is:$sig^{outer} (c_{1} ,R) = 2$,$sig^{outer} (c_{2} ,R){ = 0}$,$sig^{outer} (c_{3} ,R) = 2$,$sig^{outer} (c_{4} ,R) = 4$.The attribute with the highest significance of external attributes is $c_{4}$,then $R = R \cup \left\{ {c_{4} } \right\}$,where $NDD_{R} (U) = 2$,satisfies $NDD_{R} (U) = NDD_{C} (U)$,go to step 5.According to step 5:$\forall c \in R$,calculated the significance of internal attributes of $c$:$sig^{inner} (c_{4} ,R) \ne 0$,$sig^{inner} (c_{5} ,R) \ne 0$,then keep $R$ unchanged and output the attribute reduction set $R = \left\{ {c_{4} ,c_{5} } \right\}$ of the system $WDIS$.

## Dynamic attribute reduction algorithm for weakly labeled incomplete mixed decision systems with increased object sets

### Dynamic attribute reduction algorithm

This section outlines an incremental update mechanism for improving the efficiency of attribute reduction in a weakly labeled incomplete mixed decision system when increasing the object set. It introduces an update mechanism based on the weakly labeled relative neighborhood discernibility degree, which calculates the weakly labeled relative neighborhood discernibility degree after increasing the object set. Utilizing the original weakly labeled relative neighborhood discernibility degree, this mechanism then derives the weakly labeled relative neighborhood discernibility degree of the data after increasing the object set. As a result, the attribute reduction of the system’s properties after increasing objects was obtained. Ultimately, this section proposes a new dynamic attribute reduction algorithm for increasing the object set.

#### Theorem 2

Given a weakly labeled incomplete mixed decision system $WDIS = \left\langle {U,C \cup D,V,f} \right\rangle$,where $B \subseteq C$,$U = \left\{ {x_{1} ,x_{2} , \cdots ,x_{n} } \right\}$,$U = M \cup L$,the neighborhood radius is $\delta$. Increased an object set in the system $\Delta Y$,and the new weakly labeled incomplete mixed decision system is denoted as $WDIS{\prime} = \left\langle {U \cup \Delta Y,C \cup D,V,f} \right\rangle$,$\Delta Y = \Delta m \cup \Delta l$,where $\Delta m$ is the newly increased unlabeled object set and $\Delta l$ is the newly increased labeled object set. After increasing the object set, the incremental update formula for the weakly labeled relative neighborhood discernibility degree of $B$ under $U \cup \Delta Y$ after increasing the object set $\Delta Y$ is:

$$NDD_{B} (U \cup \Delta Y) = NDD_{B} (U) + NDD_{B} (\Delta Y) + P$$

9 $$P = 2\left( {\sum\limits_{i = 1}^{|\Delta m|} {|X_{B} (y_{i} )|} - \sum\limits_{i = 1}^{|\Delta m|} {|X_{C} (y_{i} )|} + \sum\limits_{i = 1}^{|\Delta l|} {|X_{B} (y_{i} )|} - \sum\limits_{i = 1}^{|\Delta l|} {|X_{B} (y_{i} ) \cap \left[ {y_{i} } \right]_{D} |} } \right)$$

#### Proof

Let the set of increased objects be denoted as $\Delta Y = \left\{ {y_{1} ,y_{2} , \cdots ,y_{p} } \right\}$,$Y_{B} (x)$ denotes that under set $B$,the new object $y$ belongs to the neighborhood relation of $x_{i}$,then $Y_{B} (x) = \left\{ {y \in \Delta Y|y \in NT_{B}^{\delta } (x_{i} ),x_{i} \in U} \right\}$,$X_{B} (y)$ denotes that under set $B$, the new object $x$ belongs to the neighborhood relation of $y_{i}$,then $X_{B} (y) = \left\{ {x \in U|x \in NT_{B}^{\delta } (y_{i} ),y_{i} \in \Delta Y} \right\}$.

$$\begin{gathered} NDD_{B} (U \cup \Delta Y) = NDD_{B}^{M \cup \Delta m} (C) + NDD_{B}^{L \cup \Delta l} (D) \hfill \\ = \sum\limits_{i = 1}^{|M \cup \Delta m|} {|NT_{B}^{\delta M \cup \Delta m} } (x_{i} )| - \sum\limits_{i = 1}^{|M \cup \Delta m|} {|NT_{C}^{\delta M \cup \Delta m} } (x_{i} )| + \sum\limits_{i = 1}^{|L \cup \Delta l|} {|NT_{B}^{\delta L \cup \Delta l} } (x_{i} )| - \sum\limits_{i = 1}^{|L \cup \Delta l|} {|NT_{B}^{\delta L \cup \Delta l} } (x_{i} ) \cap \left[ {x_{i} } \right]_{D} | \hfill \\ = \sum\limits_{i = 1}^{|M|} {|NT_{B}^{\delta M} (x_{i} )|} + \sum\limits_{i = 1}^{|M|} {|Y_{{\Delta m_{B} }} (x_{i} )|} + \sum\limits_{i = 1}^{|\Delta m|} {|NT_{B}^{\delta \Delta m} (y_{i} )|} + \sum\limits_{i = 1}^{|\Delta m|} {|X_{B} (y_{i} )|} - \sum\limits_{i = 1}^{|M|} {|NT_{C}^{\delta M} (x_{i} )|} - \sum\limits_{i = 1}^{|M|} {|Y_{{\Delta m_{C} }} (x_{i} )|} \hfill \\ - \sum\limits_{i = 1}^{|\Delta m|} {|NT_{C}^{\delta \Delta } m(y_{i} )|} - \sum\limits_{i = 1}^{|\Delta m|} {|X_{C} (y_{i} )|} + \sum\limits_{i = 1}^{|L|} {|NT_{B}^{\delta L} (x_{i} )|} + \sum\limits_{i = 1}^{|L|} {|Y_{{\Delta l_{B} }} (x_{i} )|} + \sum\limits_{i = 1}^{|\Delta l|} {|NT_{B}^{\delta \Delta l} (y_{i} )|} + \sum\limits_{i = 1}^{|\Delta l|} {|X_{B} (y_{i} )|} - \hfill \\ \sum\limits_{i = 1}^{|L|} {|NT_{B}^{\delta L} (x_{i} ) \cap \left[ {x_{i} } \right]_{D} |} - \sum\limits_{i = 1}^{|L|} {|Y_{{\Delta l_{B} }} (x_{i} ) \cap \left[ {x_{i} } \right]_{D} |} - \sum\limits_{i = 1}^{|\Delta l|} {|NT_{B}^{\delta \Delta l} (y_{i} ) \cap \left[ {y_{i} } \right]_{D} |} - \sum\limits_{i = 1}^{|\Delta l|} {|X_{B} (y_{i} ) \cap \left[ {y_{i} } \right]_{D} |} \hfill \\ = NDD_{B}^{M} (C) + NDD_{B}^{\Delta m} (C) + \sum\limits_{i = 1}^{|M|} {|Y_{{\Delta m_{B} }} (x_{i} )|} + \sum\limits_{i = 1}^{|\Delta m|} {|X_{B} (y_{i} )|} - \sum\limits_{i = 1}^{|M|} {|Y_{{\Delta m_{C} }} (x_{i} )|} - \sum\limits_{i = 1}^{|\Delta m|} {|X_{C} (y_{i} )|} + NDD_{{_{B} }}^{L} (D) + \hfill \\ NDD_{{_{B} }}^{\Delta l} (D) + \sum\limits_{i = 1}^{|L|} {|Y_{{\Delta l_{B} }} (x_{i} )|} + \sum\limits_{i = 1}^{|\Delta l|} {|X_{B} (y_{i} )|} - \sum\limits_{i = 1}^{|L|} {|Y_{{\Delta l_{B} }} (x_{i} ) \cap \left[ {x_{i} } \right]_{D} |} - \sum\limits_{i = 1}^{|\Delta l|} {|X_{B} (y_{i} ) \cap \left[ {y_{i} } \right]_{D} |} \hfill \\ \end{gathered}$$

Due to symmetry, there are:

$$\begin{gathered} \sum\limits_{i = 1}^{|M|} {|Y_{{\Delta m_{B} }} (x_{i} )|} = \sum\limits_{i = 1}^{|\Delta m|} {|X_{B} (y_{i} )|} ,\sum\limits_{i = 1}^{|M|} {\left| {Y_{{\Delta m_{C} }} (x_{i} )} \right| = } \sum\limits_{i = 1}^{|\Delta m|} {|X_{C} (y_{i} )|} , \hfill \\ \sum\limits_{i = 1}^{|L|} {|Y_{{\Delta l_{B} }} (x_{i} ) \cap \left[ {x_{i} } \right]_{D} |} = \sum\limits_{i = 1}^{|\Delta l|} {|X_{B} (y_{i} ) \cap \left[ {y_{i} } \right]_{D} |} ,\sum\limits_{i = 1}^{|L|} {|Y_{{\Delta l_{B} }} (x_{i} )|} = \sum\limits_{i = 1}^{|\Delta l|} {|X_{B} (y_{i} )|} \hfill \\ \end{gathered}$$

It can be concluded that:

$$\begin{gathered} NDD_{B} (U \cup \Delta Y) = NDD_{B}^{M \cup \Delta m} (C) + NDD_{B}^{L \cup \Delta l} (D) \hfill \\ = NDD_{{_{B} }}^{M} (C) + NDD_{{_{B} }}^{\Delta m} (C) + NDD_{{_{B} }}^{L} (D) + NDD_{{_{B} }}^{\Delta l} (D) \hfill \\ + 2\sum\limits_{i = 1}^{|\Delta m|} {|X_{B} (y_{i} )|} - 2|\sum\limits_{i = 1}^{|\Delta m|} {|X_{C} (y_{i} )|} + 2\sum\limits_{i = 1}^{|\Delta l|} {|X_{B} (y_{i} )|} - 2\sum\limits_{i = 1}^{|\Delta l|} {|X_{B} (y_{i} ) \cap \left[ {y_{i} } \right]_{D} |} \hfill \\ = NDD_{B} (U) + NDD_{B} (\Delta Y) + P \hfill \\ \end{gathered}$$

$$P = 2\left( {\sum\limits_{i = 1}^{|\Delta m|} {|X_{B} (y_{i} )|} - \sum\limits_{i = 1}^{|\Delta m|} {|X_{C} (y_{i} )|} + \sum\limits_{i = 1}^{|\Delta l|} {|X_{B} (y_{i} )|} - \sum\limits_{i = 1}^{|\Delta l|} {|X_{B} (y_{i} ) \cap \left[ {y_{i} } \right]_{D} |} } \right)$$

When the weak labeling incomplete mixed decision system increases the object set, the non-dynamic attribute reduction algorithm repeatedly treats the data set with the addition of new object sets as a new data set and then re-conducts attribute reduction on the data set, leading to significant time and space consumption. To address this issue, this paper proposes an incremental updating mechanism for the relative neighborhood discernibility degree of the increased object set based on Theorem 2 and introduces a dynamic attribute reduction algorithm for the added object set as shown in algorithm 2.

**Algorithm 2:**
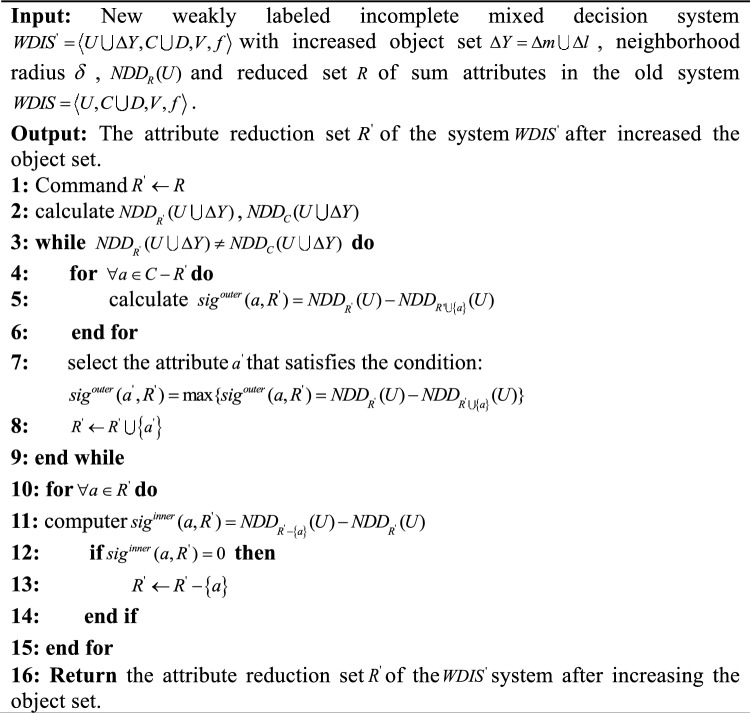
Dynamic Attribute Reduction Algorithm for Weakly labeled Incomplete Mixed Decision Systems with increased Object Set.

Time Complexity of Dynamic Algorithm 2: Step 1 calculates the relative neighborhood differentiation after updating, and its time complexity is $O(|U \cup \Delta Y||R{\prime} |)$, Step 4 gradually adds the important attribute set to the marquee attribute subset until the termination condition is satisfied, and its time complexity is $O(|U \cup \Delta Y||R{\prime} ||C - R{\prime} |)$, Step 6 removes the redundant attributes in the set of candidate attributes, and its time complexity is $O(|U \cup \Delta Y||R{\prime} |^{2} )$. Therefore, the time complexity of the dynamic Algorithm 2 is $O(|U \cup \Delta Y||R{\prime} ||C|)$, and compared to the non-dynamic Algorithm 1 which has the time complexity Algorithm 1 with the time complexity $O(|U \cup \Delta Y|^{2} |C|)^{3}$, the time complexity of the Algorithm 2 has been effectively reduced.

### Example analysis

To further illustrate the dynamic attribute reduction algorithm 2 for the incomplete weakly labeled mixed decision system proposed in this paper, the feasibility of the algorithm is validated using the original data from Table 2 and the newly increased object set as shown in Table 3. From the aforementioned analysis, it can be concluded that the attribute reduction set $R = \left\{ {c_{4} ,c_{5} } \right\}$ and $NDD_{R} (U) = NDD_{C} (U) = 2$ for system $WDIS$.

**Table 3:** Object Set Increased.

$U$	$c_{1}$	$c_{2}$	$c_{3}$	$c_{4}$	$c_{5}$	$D$
$y_{1}$	$b$	$b$	$b$	2.30	$*$	1
$y_{2}$	$a$	$a$	$b$	1.25	1.10	0
$y_{3}$	$a$	$*$	$b$	1.16	1.21	$*$

According to step 1: Command $R{\prime} = R$,calculated $NDD_{R^{\prime}} (U \cup \Delta Y) = 10$ and $NDD_{C} (U \cup \Delta Y) = 4$. According to step 2:$NDD_{{R{\prime} }} (U \cup \Delta Y) \ne NDD_{C} (U \cup \Delta Y)$, go to step 3.According to step 3:$\forall c \in C - R{\prime}$,the calculated significance of external attributes of $c$ is:$sig^{outer} (c_{1} ,R{\prime} ) = 6$,$sig^{outer} (c_{2} ,R{\prime} ) = 0$,$sig^{outer} (c_{3} ,R{\prime} ) = 4$. According to step 4: Identify the attribute in $C - R{\prime}$ with the highest significance for external attributes, denoted as $c_{1}$,then $R{\prime} = R{\prime} \cup \left\{ {c_{1} } \right\}$.According to step 5:$NDD_{{R{\prime} }} (U \cup \Delta Y) = NDD_{C} (U \cup \Delta Y) = 4$, go to step 6;According to step 6:$\forall c \in R{\prime}$,calculated the significance of internal attributes of $c$:

$$sig^{inner} (c_{1} ,R{\prime} ) \ne 0$$,$$sig^{inner} (c_{4} ,R{\prime} ) \ne 0$$,$$sig^{inner} (c_{5} ,R{\prime} ) \ne 0$$.

Thus, return the attribute reduction set $R{\prime} = c_{1} ,c_{4} ,c_{5}$ of the system $WDIS{\prime}$ after increasing the object set.

## Experimental analysis

### Experimental description

The algorithms proposed in this paper were validated using 8 data sets from the UCI^32^ database. Details of the specific datasets can be found in Table 4. The experiments were conducted on a computer with an Intel(R) Core (TM) i5-9500 CPU (3.00 GHz) and 8.0 GB of memory, running Windows 10 and using the Matlab2022a software platform.

**Table 4:** Data set table.

Number	Data set	Number of objects	Number of attributes	Number of categories
1	Lymphography	148	18	4
2	Dermatology	366	34	6
3	Hill Valley	606	100	2
4	German	1000	20	2
5	HCV	1385	28	4
6	Wave form	5000	21	4
7	Letter	20,000	16	26
8	Shuttle	43,500	9	7

In Table 4 eight datasets were subjected to min–max normalization to eliminate scale differences. In addition, to accommodate weakly labelled incomplete data types, 20% random missing values are applied to the attribute values and decision values in the dataset, so that there are 20% missing values in the attribute values and 20% missing values in the decision values. Subsequently, the datasets were subjected to attribute reduction using the Semi-D algorithm^33^, Rsfs algorithm^34^, non-dynamic algorithm 1, and dynamic algorithm 2. Since the Semi-D algorithm can only handle discrete data, a discretization process was applied to the continuous data in the datasets. To evaluate the effectiveness of these four algorithms in attribute reduction, this study utilizes the classification accuracy under the RF classifier as the evaluation metric.

### Comparison of performance of different algorithms when increasing object set

In order to verify the effectiveness of the dynamic attribute reduction algorithm 2 in augmenting the object set, the dataset was first randomly sorted to ensure that there was not only one categorical category in the extracted base data, since the dataset was initially sorted by decision value. The dataset was divided into two parts with the first 50% as the base data and the second 50% as the incremental dataset. The incremental dataset was increased in increments of 10% by adding 10 percent of the incremental dataset for the first time, 20% of the incremental dataset for the second time, and 30% of the incremental dataset for the third time, and so on for 10 iterations.

**Figure 1:**
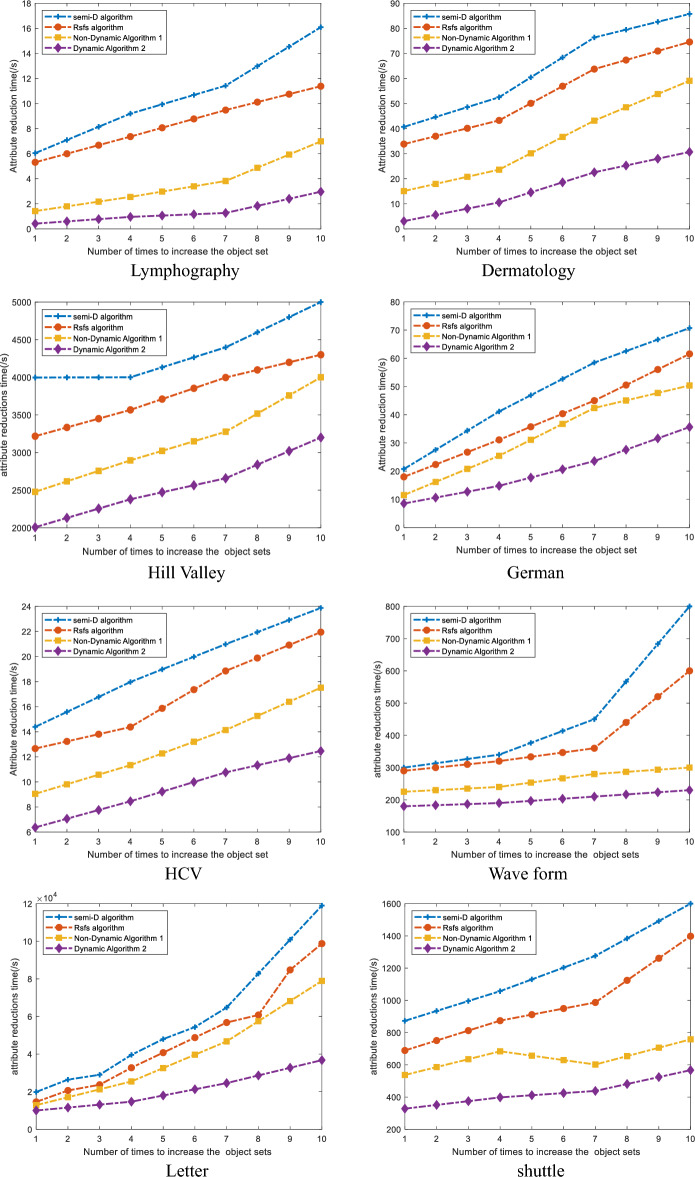
Compares the time taken for attribute reduction by four algorithms as the number of Object sets increases.

The Fig. 1 compares the time taken by the Semi-D algorithm, Rsfs algorithm, non-dynamic attribute reduction algorithm 1, and dynamic attribute reduction algorithm 2 for increasing object set attribute reduction on 8 data sets. The horizontal axis represents the number of times the object set is increased, and the vertical axis represents the time for attribute reduction, measured in seconds(s).

By observing Fig. 1, it becomes evident that the attribute reduction time of non-dynamic attribute reduction algorithm 1, dynamic attribute reduction algorithm 2, and the comparison algorithm gradually increases as the object set gradually increases in the weakly labeled incomplete mixed decision system. Notably, the attribute reduction time of dynamic attribute reduction algorithm 2 is significantly lower than that of the other three algorithms. For example, in the Dermatology dataset, when the object set was added for the tenth time, the reduction times for the Semi-D algorithm, Rsfs algorithm, non-dynamic attribute reduction algorithm 1, and dynamic attribute reduction algorithm 2 were 85.7269 s, 74.5495 s, 59.0794 s, and 30.6935 s, respectively. In comparison, the reduction times for dynamic attribute reduction algorithm 2 decreased by 64.19%, 58.82%, and 48.05%. Similarly, in the Lymphography dataset, when the object set was added for the fourth time, the reduction times for the Semi-D algorithm, Rsfs algorithm, non-dynamic attribute reduction algorithm 1, and dynamic attribute reduction algorithm 2 were 9.1935s, 7.3605 s, 2.5512 s, and 0.9562 s, respectively. The reduction times for dynamic attribute reduction algorithm 2 decreased by 89.59%, 87.01%, and 62.52%, respectively.

Based on the small data described above, the dynamic attribute reduction algorithm in this paper is also applicable to relatively large data. such as in the data Letter, when the object set was added for the 10th time, the reduction times for the Semi-D algorithm, Rsfs algorithm, non-dynamic attribute reduction algorithm 1, and dynamic attribute reduction algorithm 2 were 118,967.0 s, 98,746.0 s, 78,934.0 s, 36,786.0 s, respectively. The reduction times for dynamic attribute reduction algorithm 2 decreased by 69.08%, 62.75%, and 53.40%, respectively. Therefore, it can be clearly concluded that the attribute reduction time of the dynamic algorithm 2 in this paper is significantly reduced for both large and small datasets.

The results show that after adding the object set, Dynamic Attribute Reduction Algorithm 2 not only reduces reduction time compared to Semi-D Algorithm and Rsfs Algorithm, but also reduces reduction time significantly compared to Non-Dynamic Attribute Reduction Algorithm 1. This is because Non-Dynamic Attribute Reduction Algorithm 1, Semi-D Algorithm, and Rsfs Algorithm require recalculating the neighborhood class and weak label relative neighborhood discernibility degree of the data after increasing the object set, while Dynamic Attribute Reduction Algorithm 2 uses an incremental update mechanism to calculate the relative neighborhood discernibility degree based on the attribute reduction result of the original data set, thus reducing a significant amount of reduction time and verifying the effectiveness of Dynamic Attribute Reduction Algorithm 2 in this article.

**Figure 2:**
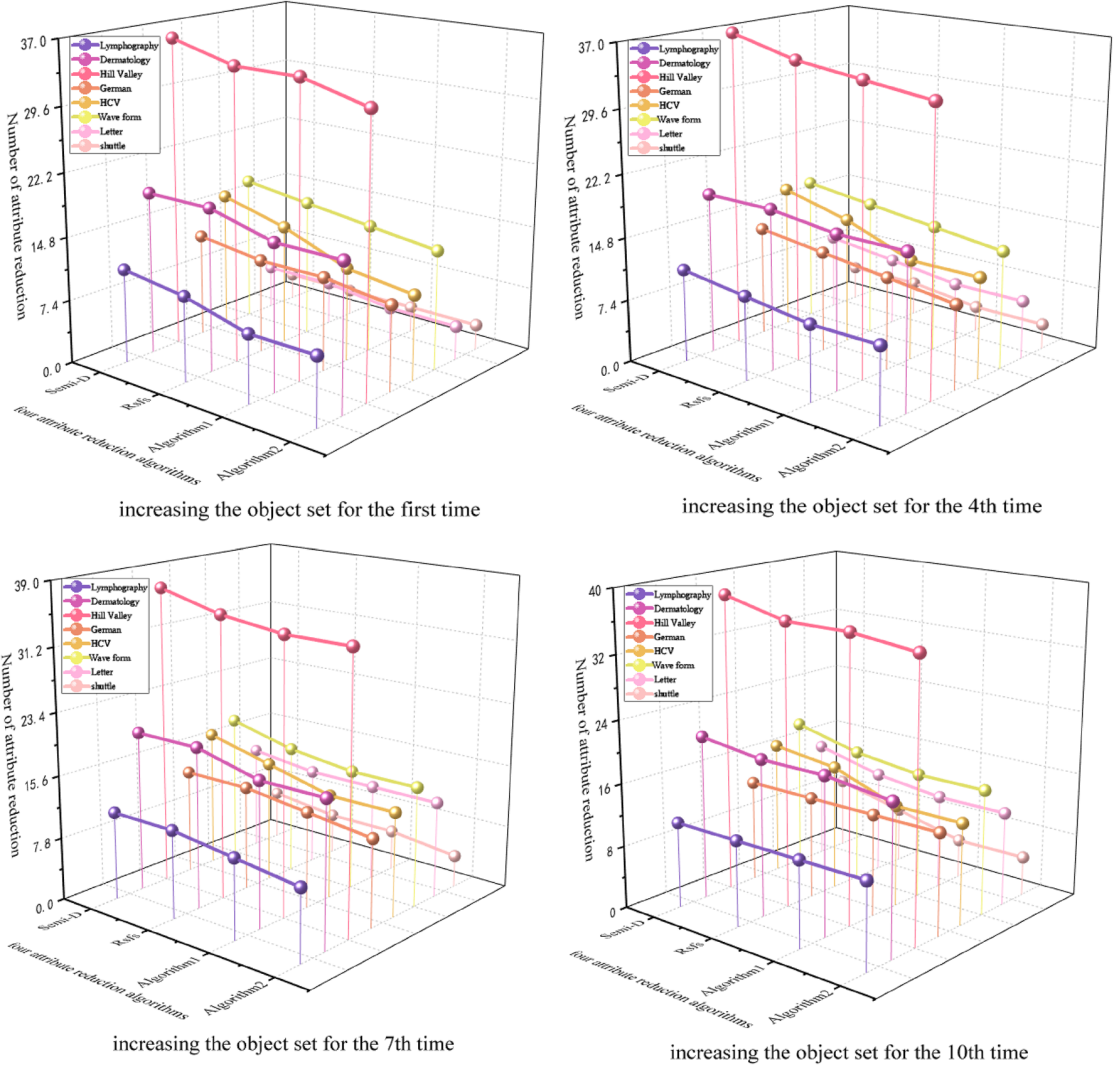
Number of attribute reduction for the four attribute reduction algorithms when increasing the set of objects.

Figure 2 presents the attribute reduction numbers for four different algorithms (Semi-D algorithm, Rsfs algorithm, non-dynamic attribute reduction algorithm 1, and dynamic attribute reduction algorithm 2) as the object sets increase for the 1st, 4th, 7th, and 10th time across 8 data sets. It is evident from the figure that the dynamic attribute reduction algorithm 2 consistently yields the smallest number of reductions, with the reduction count gradually increasing as the object set grows. Specifically, in the Dermatology data, the number of attribute reductions for the four algorithms upon increasing the object set for the first time are 19, 19, 17, and 17 respectively. For the fourth time, the numbers are 19, 19, 18, and 18. When the object set is increased for the 7th time, the count stands at 20, 20, 18, and 18, while for the 10th time, it is 21, 20, 20, and 19. In the HCV data, the number of attribute reductions for the four algorithms upon increasing the object set for the first time are 16, 14, 11, and 10 respectively. For the fourth time, the numbers are 17, 15, 12, and 12. When the object set is increased for the 7th time, the count stands at 17, 15, 13, and 13, while for the 10th time, it is 17, 16, 13, and 13. These results support the conclusion that dynamic attribute reduction algorithm 2 proves more effective compared to the other algorithms.

Table 5 presents the classification accuracies of the Semi-D algorithm, the Rsfs algorithm, non-dynamic attribute reduction algorithm 1, and dynamic attribute reduction algorithm 2 under the RF classifier for eight datasets when the object set is incremented for the 10th time. The results in Table 5 indicate that, for the 10th increase in the object set, the Dermatology and Ecoli datasets achieve the highest classification accuracy with the non-dynamic attribute reduction algorithm 1. Conversely, the German, Breast Tissue, Lymphography, Ecoli, Ionosphere, Student Performance, and HCV datasets exhibit the highest classification accuracy with the dynamic attribute reduction algorithm 2. Notably, the average classification accuracy across all datasets significantly favors dynamic algorithm 2, highlighting its superior performance. Consequently, the findings substantiate the assertion that the proposed dynamic algorithm 2 achieves higher classification accuracy Therefore, this provides a new attribute reduction algorithm with higher classification accuracy for the dynamic attribute reduction of weakly labeled incomplete mixed decision systems that increase the object set.

**Table 5:** Classification accuracy of four algorithms on the RF classifier when increasing object sets.

Data set	Semi-D algorithm	Rsfs algorithm	Non-dynamic algorithm 1	Dynamic Algorithm 2
Lymphography	0.8437	0.8764	0.8512	0.9216
Dermatology	0.7902	0.9639	0.9836	0.9758
Hill Valley	0.8593	0.9257	0.9684	0.9823
German	0.8718	0.9391	0.9427	0.9547
HCV	0.8945	0.9285	0.9758	0.9846
Wave form	0.8864	0.9153	0.9674	0.9847
shuttle	0.8734	0.9268	0.9679	0.9768
Average	0.8599	0.9251	0.9510	0.9686

## Conclusion

For the dynamic attribute reduction of weakly labeled incomplete hybrid decision systems, this paper proposes a non-dynamic attribute reduction algorithm 1 based on weakly labeled relative neighborhood discernibility degree by improving the definition of weakly labeled relative neighborhood discernibility degree by using all the unlabeled and labeled datasets, and proposes a dynamic attribute reduction algorithm 2 by constructing an incremental updating mechanism of weakly labeled relative neighborhood discernibility degree in the presence of an increased set of objects Algorithm 2. Finally, it is experimentally verified that the dynamic attribute reduction algorithm 2 proposed in this paper can significantly improve the reduction efficiency, which can obtain faster reduction time as well as higher classification accuracy, thus verifying the effectiveness of the dynamic algorithm 2 proposed in this paper, and further providing a simpler and more accurate attribute reduction algorithm for dynamic attribute reduction algorithms of weakly labeled incomplete hybrid decision-making systems. However, the data in life is not only the object set changing dynamically, but also the object set and the attribute set changing at the same time, so on the basis of the dynamic attribute reduction algorithm for increasing the object set in this paper, the next step will be to study the attribute reduction when the attribute set and the object set change at the same time in the weakly labeled incomplete hybrid decision system.

## Data Availability

The datasets generated and/or analysed during the current study are not publicly available due [REASON WHY DATA ARE NOT PUBLIC] but are available from the corresponding author on reasonable request.
